# Supplementary material for: Dietary Habits, Anthropometric Features and Daily Performance in Two Independent Long-Lived Populations from Nicoya peninsula (Costa Rica) and Ogliastra (Sardinia)
Source: Nutrients. 2020 Jun 1;12(6):1621. doi: 10.3390/nu12061621 (PMC7352961; doi:10.3390/nu12061621)
Supplement: Supplementary file 1 [file nutrients-12-01621-s001.pdf]

Table S1. *Frequencies of consumption of some typical Costa Rican foods*

**Typical Nicoyan foods**	**Female**					**Male**				
	**Never/rarely**	**2-3 servings/month**	**1-2 servings/week**	**3-5 servings/week**	**Every days**	**Never/rarely**	**2-3 servings/month**	**1-2 servings/week**	**3-5 servings/week**	**Every days**
Gallo Pinto	6.9%	0.0%	6.9%	6.9%	79.3%	3.3%	0.0 %	6.7%	3.3%	86.7%
Gallo Pinto con huevos	13.8%	3.4%	17.2%	10.3%	55.2%	13.3%	0.0%	20.0%	20.0%	46.7%
Pipian	17.2%	27.6%	27.6%	24.1%	3.4%	20.0%	20.0%	36.7%	16.3%	10.0%
Ayote	17.2%	27.6%	31.0%	17.2%	6.9%	26.7%	26.7%	33.3%	10.0%	3.3%
Tortillas de maíz	6.9%	6.9%	6.9%	10.3%	69.0%	6.7%	3.3%	6.7%	6.7%	76.7%
Perrereque	72.4%	13.8%	10.3%	3.4%	0.0%	63.3%	23.3%	6.7%	6.7%	0.0%
Pan de elote	48.3%	34.5%	13.8%	3.4%	0.0%	66.7%	20.0%	6.7%	6.7%	0.0%
Yoltamal	53.6%	28.6%	14.3%	3.6%	0.0%	53.3%	26.7%	16.7%	3.3%	0.0%
Rosquillas	24.1%	27.6%	31.0%	13.8%	3.4%	30.0%	33.3%	20.0%	13.3%	3.3%
Tanelas	31.0%	20.7%	31.0%	13.8%	3.4%	26.7%	36.7%	20.0%	16.7%	0.0%
Guiso de chilote	40.7%	22.2%	33.3%	3.7%	0.0%	41.4%	20.7%	24.1%	13.8%	0.0%
Pinolillo	44.8%	17.2%	24.1%	6.9%	6.9%	62.1%	13.8%	3.4%	13.8%	6.9
Arroz de mais	32.1%	35.7%	32.1%	0.0%	0.0%	23.3%	46.7%	26.7%	3.3%	0.0%
Flor de pinuela	92.6%	7.4%	0.0%	0.0%	0.0%	82.8%	6.9%	10.3%	0.0%	0.0%
Cuajada	20.7%	0.0%	13.8%	3.4%	62.1%	20.0%	3.3%	20.0%	6.7%	50.0%
Chilera	89.7%	0.0%	0.0%	0.0%	10.3%	76.7%	10.0%	10.0%	0.0%	3.3%
Chicha	96.3%	3.7%	0.0%	0.0%	0.0%	82.8%	6.9%	10.3%	0.0%	0.0%
Chicheme	93.1%	6.9%	0.0%	0.0%	0.0%	73.3%	13.3%	10.0%	3.3%	0.0%
Masamorra	69.0%	20.7%	10.3%	0.0%	0.0%	70.0%	20.0%	10.0%	0.0%	0.0%
Ajiaco	82.1%	14.3%	3.6%	0.0%	0.0%	89.7%	10.3%	0.0%	0.0%	0.0%
Manteca de Cerdo	88.0%	12.0%	0.0%	0.0%	0.0%	88.9%	7.4%	0.0%	0.0%	3.7%
Garrobo	85.7%	10.7%	0.0%	3.6%	0.0%	100.0%	0.0%	0.0%	0.0%	0.0%
Guaro	100.0%	0.0%	0.0%	0.0%	0.0%	95.8%	4.2%	0.0%	0.0%	0.0%
Vino de Coyol	92.0%	8.0%	0.0%	0.0%	0.0%	96.2%	3.8%	0.0%	0.0%	0.0%

Table S2. *Frequencies of consumption of some typical Ogliastrine foods.*

**Common foods**	**Female**					**Male**				
	**Never/rarely**	**2-3 servings/month**	**1-2 servings/week**	**3-5 servings/week**	**Every days**	**Never/rarely**	**2-3 servings/month**	**1-2 servings/week**	**3-5 servings/week**	**Every days**
Pistokku bread[a]	0.00%	0.00%	0.00%	0.00%	100.00%	0.00%	0.00%	0.00%	0.00%	100.00%
Minestrone[b]	56.30%	31.30%	12.50%	0.00%	0.00%	57.90%	31.60%	5.30%	5.50%	0.00%
Culurgiones[c]	18.00%	21.30%	37.10%	20.20%	3.40%	8.20%	24.60%	47.50%	18.00%	1.60%
Fava and lard[d]	7.90%	49.40%	36.00%	6.70%	0.00%	3.30%	63.90%	26.20%	6.60%	0.00%
Seadas with honey[e]	2.20%	57.30%	39.30%	1.10%	0.00%	1.60%	60.70%	36.10%	1.60%	0.00%
Turredda cun curcuriga[f]	13.50%	46.10%	40.40%	0.00%	0.00%	3.30%	60.70%	36.10%	0.00%	0.00%
Gathulis[g]	7.90%	41.60%	40.40%	10.10%	0.00%	3.30%	19.70%	60.70%	16.40%	0.00%
Casu ajedu[h]	5.60%	14.60%	32.60%	44.90%	2.20%	4.90%	6.60%	32.80%	50.80%	4.90%
Goat mature cheese	6.70%	15.70%	27.00%	48.30%	2.20%	4.90%	6.60%	27.90%	52.50%	8.20%
Goat milk/yoghurt	3.40%	30.30%	48.30%	10.10%	7.90%	4.90%	18.00%	14.80%	31.10%	31.10%
Peas and potatoes	0.00%	55.10%	32.60%	12.40%	0.00%	0.00%	50.80%	27.90%	21.30%	0.00%
Roasted piglet	7.90%	52.80%	30.30%	9.00%	0.00%	3.30%	44.30%	39.30%	13.10%	0.00%
Cannonau red wine[i]	2.20%	27.00%	22.50%	28.10%	20.20%	3.30%	14.80%	16.40%	24.60%	41.00%
Myrtle liqueur[j]	6.70%	42.70%	39.30%	11.20%	0.00%	3.30%	18.00%	42.60%	32.80%	3.30%

[a]crisp, thin flat bread of wheat; [b]legumes soup with potatoes, cabbages and zucchini; [c]pasta filled with cheese, flour and mashed potatoes; [d]broad beans with pork lard and potatoes; [e]fried puff pastry stuffed with cheese and garnished with honey and lemon; [f]focaccia with zucchini, bacon and onion; [g]potato and cheese ring fritters; [h]semi-solid sour cheese; [i]red wine with a high polyphenol content; [j]sweet liqueur extracted from the berries of *Myrtus communis*.

Table S3. Correlation between frequency of food consumption and anthropometric measurements in the elderly of Nicoya and Ogliastra

	**Nicoya**						
	**Body weight**	**Body height**	**BMI**	**Knee-floor distance**	**Waist circumference**	**Average arm circumference**	**Average calf circumference**
Meat	0.140	0.063	–0.010	0.029	0.096	0.139	–0.024
Fish	0.075	0.102	–0.038	–0.004	0.005	0.050	0.002
Legumes	0.019	0.068	–0.120	–0.053	0.207	0.052	–0.065
Salad	0.094	0.108	–0.085	0.162	0.044	0.080	0.034
Cereals	0.105	0.026	0.101	–0.077	0.179	0.222	0.122
Pasta	–0.081	0.127	–0.062	0.026	–0.171	0.053	–0.105
Potato	0.009	–0.062	0.059	–0.086	0.017	0.036	–0.066
Fruit	0.075	0.104	0.022	0.052	0.160	0.074	0.012
Sweets	0.002	0.107	–0.051	0.212	–0.045	0.067	0.118
Dairy food, except milk	0.012	–0.082	0.048	–0.105	0.066	–0.002	–0.096
Milk	0.059	0.099	0.082	0.006	0.107	0.114	0.214
Coffee	0.123	–0.019	0.026	0.033	0.145	0.106	0.016

	**Ogliastra**						
	**Body weight**	**Body height**	**BMI**	**Knee-floor distance**	**Waist circumference**	**Average arm circumference**	**Average calf circumference**
Meat	**0.330** *	0.105	0.201	0.247	0.066	**0.466** **	**0.513** **
Fish	0.139	–0.044	0.010	0.016	0.115	0.314	0.231
Legumes	–0.075	–0.058	0.040	0.001	0.177	–0.113	–0.053
Salad	0.058	0.042	0.088	0.134	0.071	–0.205	–0.060
Cereals	0.050	0.102	–0.167	0.210	0.228	–0.041	0.062
Pasta	–0.044	0.067	0.024	0.105	–0.059	–0.035	0.017
Potato	0.071	0.106	–0.030	–0.090	**0.373** *	0.016	0.258
Fruit	0.059	0.004	–0.014	–0.077	–0.025	0.052	0.169
Sweets	–0.040	–0.098	–0.101	–0.049	–0.085	0.045	–0.127
Dairy food, except milk	0.008	0.001	0.080	0.071	0.088	–0.129	0.127
Milk	–0.051	0.084	–0.058	0.233	0.008	**–0.338** *	–0.147
Coffee	0.081	–0.052	0.016	0.026	0.098	0.112	0.048
